# 654. Assessment of Patient Attitudes Towards Teleconsultation for Burn Injury

**DOI:** 10.1093/jbcr/irag033.491

**Published:** 2026-04-06

**Authors:** Grace Roth, Victoria R Hammond, Diane Gillis, Matthew Bozeman

**Affiliations:** University of Louisville School of Medicine, Louisville, KY; University of Louisville Burn Center, Louisville, KY; University of Louisville Burn Center, Louisville, KY; University of Louisville Burn Center, Louisville, KY

## Abstract

**Introduction:**

Regionalization of burn care has led to opportunities for telemedicine utilization. Challenges exist in remote burn care management, and previously patient preference was not frequently assessed. This study sought to assess patients’ attitudes regarding teleconsultation for initial burn evaluation.

**Methods:**

A burn teleconsultation program was developed between an ABA verified burn center and a regional hospital 106 miles away. This single regional center was chosen to control for the effect of distance from burn center. The burn center’s Trauma Database was queried for patients who received teleconsultation or were transferred from this regional hospital between January 2020 and December 2024. 58 patients were identified meeting inclusion criteria. Mailers and phone calls were used to attempt to contact all 58 patients, with a phone-based questionnaire used for scoring. 5-point Likert scale was used to assess patient attitudes. 11 patients (19.0%) consented to participation and completed a survey. Statistical analysis of results was performed using STATA.

**Results:**

Demographic information is in Table 1. When asked if participants would prefer to receive a teleconsultation prior to transfer, only 2 (18.2%) disagreed. 9 (81.8%) patients agreed or were neutral. When asked if participants would prefer to have a family member receive a teleconsultation prior to transfer, 2 (18.2%) disagreed. 7 (63.6%) patients agreed or strongly agreed. However, when asked if patients felt that it was a “waste of their time” to be transferred without a teleconsultation, attitudes were mixed. 3 patients (27.3%) strongly disagreed, 3 (27.3%) patients were neutral, and 2 patients (18.2%) strongly agreed. Patients who received a teleconsultation had more positive attitudes towards teleconsultation than those who did not.

**Conclusions:**

This data suggests that patient attitudes towards teleconsultation are relatively positive, especially amongst patients who received a teleconsultation.

**Applicability of Research to Practice:**

This data supports patient interest in continued expansion of teleconsultation programs for burn injury. Telemedicine continues to be a viable option for regionalized care, despite current challenges.

**Funding for the study:**

N/A.

